# Mice with *Dab1* or *Vldlr* insufficiency exhibit abnormal neonatal vocalization patterns

**DOI:** 10.1038/srep25807

**Published:** 2016-05-17

**Authors:** E. R. Fraley, Z. D. Burkett, N. F. Day, B. A. Schwartz, P. E. Phelps, S. A. White

**Affiliations:** 1 Molecular, Cellular and Integrative Physiology Graduate Program, University of California, Los Angeles, USA; 2 Department of Integrative Biology and Physiology, University of California, Los Angeles, USA; 3 Undergraduate Interdepartmental Program in Neuroscience, University of California, Los Angeles, USA.

## Abstract

Genetic and epigenetic changes in components of the Reelin-signaling pathway (*RELN, DAB1*) are associated with autism spectrum disorder (ASD) risk. Social communication deficits are a key component of the ASD diagnostic criteria, but the underlying neurogenetic mechanisms remain unknown. *Reln* insufficient mice exhibit ASD-like behavioral phenotypes including altered neonatal vocalization patterns. Reelin affects multiple pathways including through the receptors, Very low-density lipoprotein receptor (Vldlr), Apolipoprotein receptor 2 (Apoer2), and intracellular signaling molecule Disabled-1 (Dab1). As Vldlr was previously implicated in avian vocalization, here we investigate vocalizations of neonatal mice with a reduction or absence of these components of the Reelin-signaling pathway. Mice with low or no Dab1 expression exhibited reduced calling rates, altered call-type usage, and differential vocal development trajectories. Mice lacking Vldlr expression also had altered call repertoires, and this effect was exacerbated by deficiency in Apoer2. Together with previous findings, these observations 1) solidify a role for Reelin in vocal communication of multiple species, 2) point to the canonical Reelin-signaling pathway as critical for development of normal neonatal calling patterns in mice, and 3) suggest that mutants in this pathway could be used as murine models for Reelin-associated vocal deficits in humans.

Reelin is a large secreted glycoprotein that has numerous nervous system functions including regulating neuronal migration, neuronal excitability, and dendritic morphology^1^,^2^,^3^,^4^,^5^,^6^. Murine *reeler* mutants (*Reln*^*−*/*−*^) do not express Reelin and exhibit a characteristic phenotype of a reeling gait, disorganization of laminated structures including the neocortex, cerebellum, and hippocampus, and a reduction in cerebellar volume^1^,^7^,^8^,^9^,^10^. When Reelin binds to Very low-density lipoprotein receptor (Vldlr) and/or Apolipoprotein receptor 2 (Apoer2), this initiates binding of Disabled-1 (Dab1) to the internal domain of the receptors^11^,^12^. Dab1 is then phosphorylated at critical tyrosine residues by Src-family kinases to influence a wide array of downstream effectors^6^,^13^,^14^.

*RELN* has been identified as a risk allele for autism spectrum disorder (ASD) in multiple populations^15^,^16^,^17^,^18^,^19^,^20^,^21^,^22^,^23^. Polymorphisms throughout *RELN* include variants in both coding and non-coding regions. Changes leading to an expansion in GGC repeats in the 5′ region reduce *RELN* expression levels and confer ASD risk in some cases^24^. Reelin protein (RELN) is low in post-mortem brain tissue of ASD patients compared to controls^25^. Additionally, *RELN* mRNA is low in the cerebellum and cortex of these patients^25^. Epigenetic down regulation of *RELN* via increased methylation of its promoter is also linked to increased ASD risk^26^. Intriguingly, *DAB1* polymorphisms are associated with ASD risk in the Chinese-Han population whereas *RELN* polymorphisms are not^27^. These results indicate that not only *RELN*, but the function of the downstream Reelin-signaling pathway could be involved in the etiology of ASD.

Social communication deficits are a key diagnostic feature of ASD. Autistic symptoms are generally undetected at birth, but instead appear over time and reflect differential developmental trajectories^28^. High risk infants, i.e. children with one or more ASD siblings, and infants later diagnosed with ASD, have altered acoustic features of their cries^29^. At 6 months, their cries are more disordered and of higher pitch compared with those of typically developing children. High risk infants also exhibit abnormal pre-linguistic vocal behavior such as making fewer speech-like vocalizations and more non-speech vocalizations as well as producing fewer consonant types than typically developing peers^30^. Given these observations, examination of the amount and acoustic parameters of infant cries could serve as a tool for early ASD detection.

Genetic causes are linked to 10–25% of ASD cases^31^. Investigation of how these gene mutations alter behavioral phenotypes, and the underlying brain organization and function, are enabled by mouse models^32^. Neonatal mouse pups typically emit ultrasonic vocalizations (USVs) when isolated from the dam which act as a signal for the dam to retrieve and care for the pups^33^. Pups are entirely reliant on the dam during this time (P0-P14), and thus appropriate communication cues are critical to their survival. Isolation USVs first occur at ~postnatal day 4 (P4) and peak around P6-7, before gradually declining at P14 when the pup is able to self-retrieve^34^. Dams preferentially retrieve pups that call more and prefer more elaborate call types^35^,^36^. Reductions in total calling, delays in the peak calling age, and an altered call repertoire occur in many mouse ASD models^37^,^38^,^39^,^40^,^41^,^42^,^43^,^44^,^45^. Murine neonatal isolation calls are considered to be more like human baby cries than early speech. Although laboratory mice are not robust vocal learners^46^, their vocal behavior can reflect sociability and mechanisms of communication that subserve both learned and unlearned vocalizations.

Because humans with ASD were reported to have low levels of Reelin, the *Reln*^+/−^ mouse was proposed as a model for ASD^47^. *Reln*^+/−^ mice have a 50% reduction in Reelin protein and lack the typical neuronal migration deficits seen in *Reln*^*−*/*−*^ mice^48^. *Reln*^+/−^ mice however, exhibit GAD67 down-regulation in the frontoparietal cortex^49^, Purkinje cell loss and hypoplasia of the cerebellum^50^ and parvalbumin-positive cell loss in the striatum^51^, resulting in changes to cortico-striatal plasticity^50^. These changes are parallel to those in human ASD cases which show the following abnormalities: low GAD67 across brain regions including the frontal cortex^52^; Purkinje cell loss and reduced cerebellar volume^53^,^54^, and altered connectivity and function of the striatum^55^,^56^. Genetic vulnerability can determine phenotype by interacting with the environment; other studies have examined multivariate conditions (separation, stress, drug/pesticide exposure) that interact with the reduced Reln expression to mirror ASD-like phenotypes^57^,^58^,^59^,^60^,^61^.

To investigate whether or not Reelin deficiency alone creates an ASD-like phenotype, the early vocal behavior of the *Reln*^+/−^ and *Reln*^*−*/*−*^ mice was characterized^57^,^62^. *Reln*^+/−^ mice show a delay in the age of peak isolation USV calling, whereas *Reln*^*−*/*−*^ mice have low calling rates at all measured time points (P2-P12), most likely due to gross motor deficits^57^. Repertoires of P6 pups are altered in a gene-dose dependent manner, with a particularly large expansion of two-syllable call types (see Methods for call type classifications)^62^. Differences in repertoire based on genotype disappear as pups mature (P8-12). These findings indicate a deficit in early vocal communication in *Reln*^+/−^ mice.

*VLDLR* is a known target of the language-associated transcription factor *FOXP2* in humans^63^. Moreover, vocally regulated gene networks in the zebra finch basal ganglia (Area X)^64^ include *Vldlr, Dab1,* and *Reelin*; *Vldlr* is in the same gene module as *FoxP2*. These observations suggest that the Reelin-signaling pathway is essential for normal vocal development in multiple species.

Here, we examine the early vocal phenotypes of mice with reductions in *Vldlr, Apoer2* and *Dab1*. Findings are then compared with those from *Reln*^+/−^ and *Reln*^*−*/*−*^ mice^62^ in order to attribute changes in vocal development to the canonical Reelin-signaling pathway. Given the greater incidence of ASD in the male population^65^, sex as a contributing factor was also examined.

## Results

### Amount of calling depends on *Dab1* genotype at P7 but not at P14

**Figure 1:**
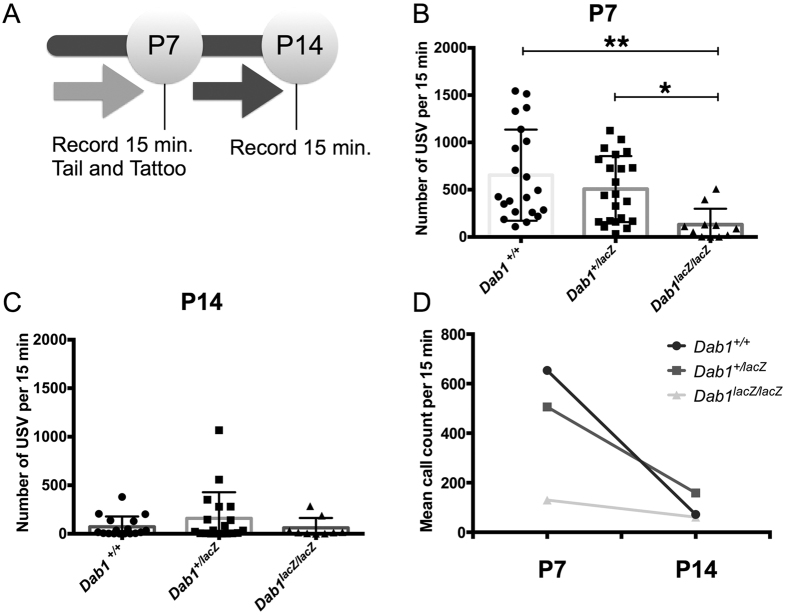
*Dab1* genotype and postnatal age affect pup isolation call amounts. (**A**) Experimental paradigm. (**B**) At P7, *Dab1* mice exhibit gene-dose dependent effects on call number: wild-type pups (*Dab1*^+/+^) call the most, followed by heterozygote pups (*Dab1*^+/*lacZ*^), while homozygous mutant pups (*Dab1*^*lacZ*/*lacZ*^) call the least (**p = *0.0001*; *p = *0.0002*). (**C**) At P14, no differences between call rates are observed. (**D**) Developmental trajectories in calling amount vary by genotype. Between P7 and P14, *Dab1*^+/+^ pups exhibit the steepest decline (**p < 0.0001) followed by *Dab1*^+/*lacZ*^ pups (*p = *0.0007*). Call rate did not decline in *Dab1*^*lacZ*/*lacZ*^ mice (*p* = *0.297*, NS).

To test for an early social communication deficit, *Dab1* deficient mouse pups were recorded (Fig. 1A). The number of calls produced by male and female *Dab1* pups of each of the 3 genotypes was quantified (*Dab1*^+/+^ N = 21, *Dab1*^+/*lacZ*^ N = 23, *Dab1*^*lacZ*/*lacZ*^ N = 11). A significant effect of genotype on calling behavior was observed at P7, with no effect of sex (Fig. 1B; two-way ANOVA, sex effect *p* = *0.308*, genotype effect *p* = *0.005*, interaction *p* = *0.671*). As call number did not differ based on sex, data from both sexes were pooled (Supplemental Fig. 1A). At P7, *Dab1*^+/+^ mice called the most and their call counts were significantly greater than those of the *Dab1*^*lacZ*/*lacZ*^ mice (t-test; *p* = *0.0001*). The *Dab1*^+/*lacZ*^ mice called more than the *Dab1*^*lacZ*/*lacZ*^ mice (t-test, *p* = *0.004*). The *Dab1*^*lacZ*/*lacZ*^ mutants made the least number of calls at this time point, a result that may reflect their severe motor deficits. Thus, at P7, a *Dab1* gene-dose dependent effect on calling amount was evident, with no effect of sex.

At P14, the amount of calling was relatively low and similar across all three genotypes (Fig. 1C; *Dab1*^+/+^ N = 17, *Dab1*^+/*lacZ*^ N = 19, *Dab1*^*lacZ*/*lacZ*^ N = 9; two-way ANOVA; genotype effect *p* = *0.545*, sex effect *p* = *0.400*, interaction *p* = *0.296,* NS). The amount of calling by *Dab1*^+/+^ mice did not differ from *Dab1*^+/*lacZ*^ mice (t-test, *p* = *0.206*, NS) or *Dab1*^*lacZ*/*lacZ*^ mice (t-test, *p* = *0.800,* NS). Interestingly, comparison of the total call counts between P7 and P14 time points by genotype reveals a differential rate of age-related decline (Fig. 1D; two-way ANOVA; age effect *p* = *0.0001*, genotype effect *p* = *0.006*, interaction *p* = *0.0143*). The *Dab1*^+/+^ mice exhibit a steep fall-off in calling amount between P7 and P14 (t-test*, p* < *0.0001*) in line with the normal developmental decline of isolation calling^34^. Comparatively, in *Dab1*^+/*lacZ*^ mice the decline from P7 to P14 was less significant (t-test, *p* = *0.001*); and the *Dab1*^*lacZ*/*lacZ*^ mice did not significantly differ in the amount of calling between P7 and P14 (t-test, *p* = *0.275*). These findings indicate altered vocal developmental trajectories for the *Dab1* reduced (*Dab1*^+/*lacZ*^) and null (*Dab1*^*lacZ*/*lacZ*^) mice.

### *Dab1* genotype affects P7 call repertoires

**Figure 2:**
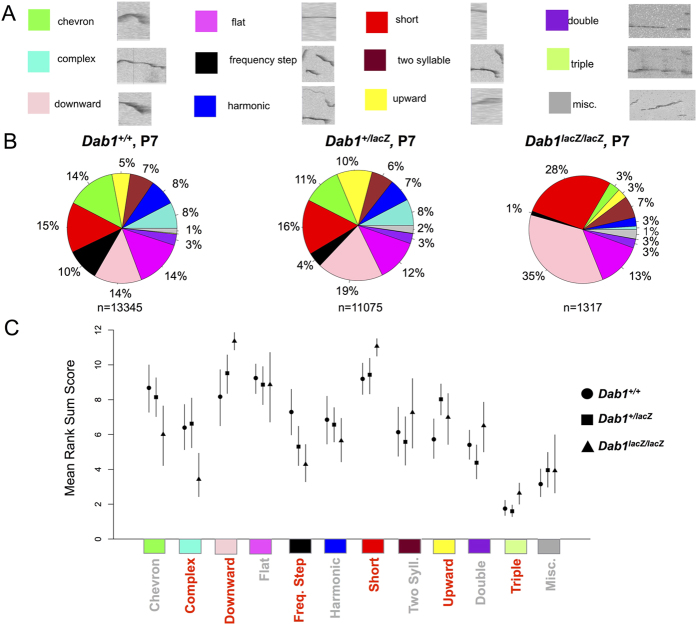
*Dab1* genotype affects P7 call repertoire. (**A**) Representative syllables for each call cluster, known as eigen syllables, are shown with their classifications and representative colors. (The same colors are used in Figs 3,5 and 6) (**B**) Pie charts depict P7 calling repertoires of *Dab1*^+/+^ (N = 13,345 calls from 21 pups), *Dab1*^+/*lacZ*^ (N = 11,075 calls from 22 pups), and *Dab1*^*lacZ*/*lacZ*^ mice (N = 1,317 calls from 7 pups). (**C**) Quantitative repertoire analysis. Data are rank sum transformed such that 12 on the y axis denotes high call use probability and 1, low call use probability. Lines indicate 95% confidence intervals, shapes correspond to genotypes: Circle (*Dab1*^+/+^), square (*Dab1*^+/*lacZ*^), and triangle (*Dab1*^*lacZ*/*lacZ*^). Significant differences are indicated when call categories are highlighted in red on the x axis, and the 95% confidence intervals do not overlap between one or more genotypes. Differences are found for the following categories: complex, downward, frequency step, short, upward, and triple.

Next, the types of calls were analyzed to determine any genotype-dependent differences (Fig. 2A). In addition to an altered amount of calling, described above, the types of calls were also altered in a gene dose-dependent manner (Fig. 2B,C). As with call number, the call repertoire exhibited a great deal of variability between pups, even within the same genotype (Supplemental Fig. 2A,B). To enable comparison, data were normalized to create pie charts depicting the combined call repertoire for each genotype (See Methods; Fig. 2B). At P7, *Dab1*^+/+^ mice (N = 13,345 calls from 21 mice) had a relatively diverse repertoire. When comparing calls from *Dab1*^+/+^ and *Dab1*^+/*lacZ*^ pups (N = 11,075 calls, from 22 mice), *Dab1*^+/*lacZ*^ had significantly more upward call types. Otherwise, *Dab1*^+/*lacZ*^ mice had an intermediate phenotype. Trends that placed the *Dab1*^+/*lacZ*^ between the *Dab1*^+/+^ and *Dab1*^*lacZ*/*lacZ*^ mice include an intermediate level of the downward and frequency step call types. The *Dab1*^*lacZ*/*lacZ*^ pups (N = 1,317 calls, from 7 mice) exhibited a relatively restricted repertoire comprised of significantly more short and downward calls than found in the other genotypes. The *Dab1*^+/+^ pups made significantly more complex and frequency step calls than did the *Dab1*^*lacZ*/*lacZ*^ mice. *Dab1*^*lacZ*/*lacZ*^ pups also had significantly more of an unusual call type, the triple, than the other groups. Chevron, flat, harmonic, two-syllable, double, and miscellaneous call types did not differ significantly based on genotype. A few sex differences in repertoire at P7 were found for *Dab1*^*lacZ*/*lacZ*^ (Supplemental Fig. 1C). However, based on the lack of sex effect on repertoire in *Dab1*^+/*lacZ*^ (Supplemental Fig. 1B), call type data were pooled across sexes to provide greater power for further analysis. Notably, repertoire analysis of all wild-type mice across experiments pooled revealed no repertoire differences based on sex (Supplemental Fig. 3).

**Figure 3:**
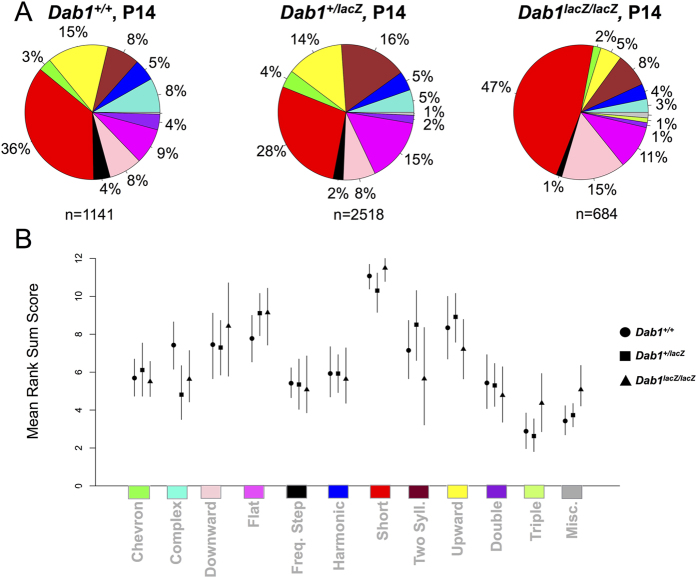
*Dab1* genotype does not affect P14 call repertoire. (**A**) Call repertoires for wild-type (N = 1,141 calls from 11 pups), heterozygous (N = 2,518 calls from 19 pups), and homozygous mice (N = 684 calls from 3 pups). (**B**) For each genotype, quantification shows 95% confidence intervals resampled about the median call usage. Shapes correspond to genotypes: circle (*Dab1*^+/+^), square (*Dab1*^+/*lacZ*^), and triangle (*Dab1*^*lacZ*/*lacZ*^). There are no significant differences.

At P14, *Dab1*^+/+^ and *Dab1*^+/*lacZ*^ repertoires were fairly similar, while those of *Dab1*^*lacZ*/*lacZ*^ mice appeared more restricted than *Dab1*^+/+^ (Fig. 3A, Supplemental Fig. 2B). There were however, no statistically significant differences in call repertoires (Fig. 3B; syllables analyzed: *Dab1*^+/+^ N = 1,141 from 13 mice, *Dab1*^+/*lacZ*^ N = 2,518 from 13 mice, *Dab1*^*lacZ*/*lacZ*^ N = 684 from 7 mice). The lack of statistical significance is likely due to the relatively low numbers of calls made at this time point, especially by *Dab1*^*lacZ*/*lacZ*^ mice. In summary, at P7, *Dab1*^+/*lacZ*^ and *Dab1*^*lacZ*/*lacZ*^ mice exhibited partially and extremely restricted call repertoires, respectively, relative to the *Dab1*^+/+^ mice. *Dab1*^*lacZ*/*lacZ*^ repertoires included a decreasing level of some of the more elaborate call types and an increase in some of the simpler ones. Notably, despite gross motor deficits, *Dab1*^*lacZ*/*lacZ*^ pups were able to make a majority of the call types described. Thus, changes in their repertoires may not be fully attributable to global motor deficits.

### Effect of *Vldlr* ablation on calling rates at P7 and P14

**Figure 4:**
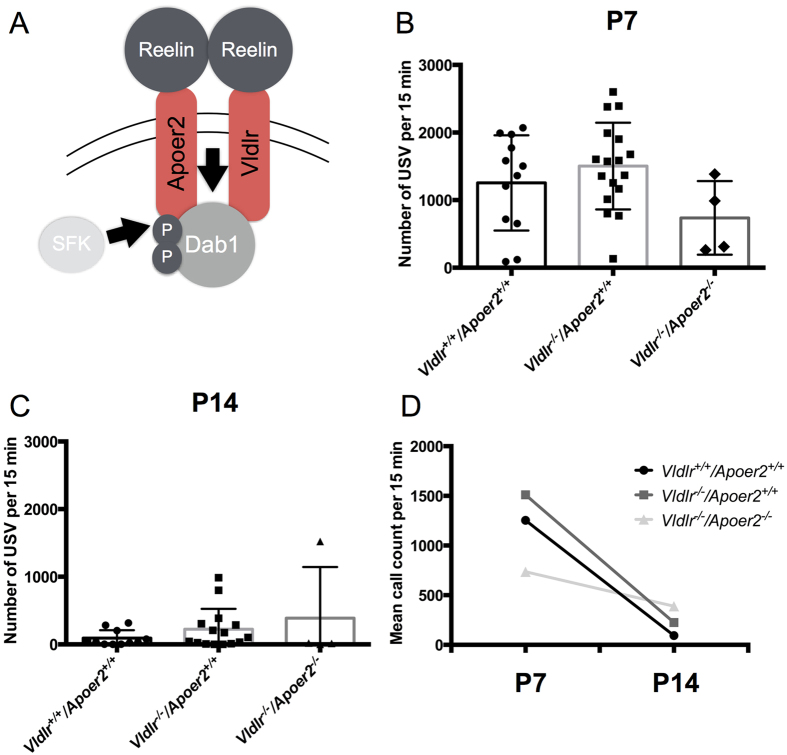
*Vldlr* and *Vldlr/Apoer2* insufficient pups have altered developmental trajectories in calling amount. (**A**) Schematic depicts the canonical Reelin-signaling pathway. Signal is transduced via Reelin binding to receptors *Vldlr* and *Apoer2* to initiate phosphorylation of *Dab1* via Src-family kinases. (**B**) Quantification of P7 call counts from wild-type pups (*Vldlr*^+/+^*/Apoer2*^+/+^, N = 12), *Vldlr* single receptor mutants (*Vldlr*^*−*/*−*^*/Apoer2*^+/+^; N = 18) and double receptor mutants (*Vldlr*^*−*/*−*^*/Apoer2*^*−*/*−*^; N = 4). Trends suggest that the double receptor mutants call less than the other genotypes. (**C**) Quantification of P14 call counts for pups of all three genotypes reveal no significant differences. (**D**) Developmental trajectories between P7 and P14 differ by genotype. Call amounts of *Vldlr*^+/+^*/Apoer2*^+/+^ pups (*p* = *0.0001*) and *Vldlr*^*−*/*−*^*/Apoer2*^+/+^ pups decline (**p < *0.0001*) but those of *Vldlr*^*−*/*−*^*/Apoer2*^*−*/*−*^ pups do not (*p* = *0.486*, NS).

Vldlr and Apoer2 are high-affinity Reelin receptors essential for transduction of the signal to Dab1 (Fig. 4A). To further test that vocal deficits could be related to *Vldlr* insufficiency, we examined the effect of *Vldlr* deletion with or without *Apoer2*. Wild-type (*Vldlr*^+/+^*/Apoer2*^+/+^; N = 12), *Vldlr* single receptor mutants (*Vldlr*^*−*/*−*^*/Apoer2*^+/+^; N = 18), and *Vldlr/Apoer2* double receptor mutants (*Vldlr*^*−*/*−*^*/Apoer2*^*−*/*−*^; N = 4) were recorded at P7 and P14. At P7, there were no significant differences in the number of calls emitted by each group and no effect of sex (Fig. 4B, Supplemental Fig. 4A; two-way ANOVA, genotype effect *p* = *0.226*, sex effect *p* = *0.447*, interaction *p* = *0.700,* NS). This lack of genotype effect on call amount at P7 could be due to the low number of double mutants obtained for recording (N = 4). Upon closer examination, the call counts of *Vldlr*^*−*/*−*^*/Apoer2*^*−*/*−*^ mice (mean call count = 736.5) were close to statistical significance as being lower than those of the *Vldlr*^*−*/*−*^*/Apoer2*^+/+^ mice (mean call count = 1504; t-test *p* = *0.057*).

At P14, there were no differences in calling amount based on the *Vldlr* or *Apoer2* genotype, but there was a sex difference (two-way ANOVA, genotype effect *p* = *0.325*, sex effect *p* = *0.010*, interaction *p* = *0.224*). The males appear to be more adversely affected and called less than the females (Supplemental Fig. 4A). Developmental trajectories of each group were then examined (two-way ANOVA; genotype effect *p* = *0.220*, age effect *p* = *0.001*, interaction *p* = *0.085*). There was a significant decrease in calling from P7 to P14 (Fig. 4D) by the *Vldlr*^+/+^*/Apoer2*^+/+^ (t-test *p* = *0.0001*) and *Vldlr*^*−*/*−*^*/Apoer2*^+/+^ mice (t-test *p* < *0.0001*). *Vldlr*^*−*/*−*^*/Apoer2*^*−*/*−*^ mice, much like the *Dab1*^*lacZ*/*lacZ*^ mice, did not have a significant difference in call counts between P7 and P14 (t-test *p* = *0.459,* NS). The normal developmental decline in calling rate was observed for both *Vldr*^+/+^*/Apoer2*^+/+^ and *Vldlr*^*−*/*−*^*/Apoer2*^+/+^ mice and but not for *Vldlr*^*−*/*−*^*/Apoer2*^*−*/*−*^ mice.

### *Vldlr/Apoer2* genotype affects call repertoires at P7 and P14

**Figure 5:**
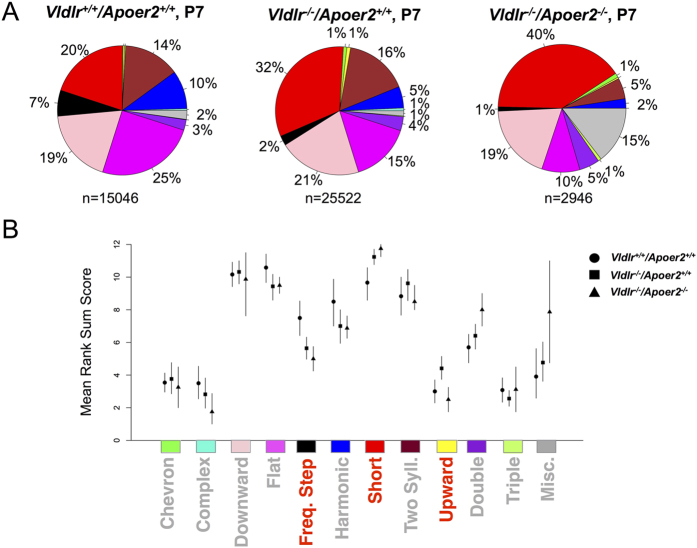
P7 call repertoire is influenced by *Vldlr*/*Apoer2* genotype. Repertoires were determined and are depicted as in Fig. 2. Mice of *Vldlr*^+/+^*/Apoer2*^+/+^ (N = 15,046 calls from 12 pups), *Vldlr*^*−*/*−*^*/Apoer2*^+/+^ (N = 25,522 calls from 18 pups), and *Vldlr*^*−*/*−*^*/Apoer2*^*−*/*−*^ genotypes (N = 2,946 from 4 pups) exhibit increasingly restricted repertoires, respectively. (**B**) Quantification of calling repertoire differences between genotypes. Each shape corresponds to a genotype: Circle signifies *Vldlr*^+/+^*/Apoer2*^+/+^; square *Vldlr*^*−*/*−*^*/Apoer2*^+/+^, triangle *Vldlr*^*−*/*−*^*/Apoer2*^*−*/*−*^. Significant differences (highlighted in red) were found for the following call types: frequency steps, short and upward.

**Figure 6:**
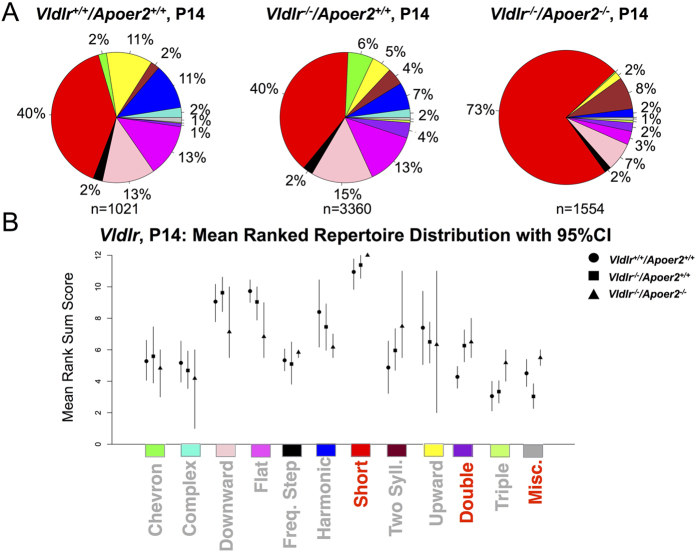
P14 call repertoire is influenced by *Vldlr*/*Apoer2* genotype. (**A**) Pie charts depict call repertoires of *Vldlr*^+/+^*/Apoer2*^+/+^ (N = 1,021 calls from 9 pups), *Vldlr*^*−*/*−*^*/Apoer2*^+/+^ (N = 3,360 from 13 pups) and *Vldlr*^*−*/*−*^*/Apoer2*^*−*/*−*^ mice (N = 1,554 calls from 3 pups). (**B**) Repertoire analysis shows significant differences in the short, double and miscellaneous categories as revealed by non-overlapping confidence intervals.

The call repertoire based on presence of *Vldlr* was then assessed at P7 and P14 (Figs 5 and 6). Despite the high degree of individual variability, there were significant differences in call usage that paralleled what was observed for *Dab1* mice (Fig. 5B; Supplemental Fig. 2C,D). To improve power, data were pooled across sex, as only minimal sex differences were observed (Supplemental Fig. 4B,C). Overall, the severity of receptor deficiency was inversely related to the call repertoire, with greater deficiencies corresponding to more restricted repertoires (Fig. 5A). At P7, the *Vldlr*^+/+^*/Apoer2*^+/+^ pups (N = 15046 calls) made more frequency step calls and fewer short calls compared to the other groups. There were significantly more short calls and fewer frequency step calls in both *Vldlr*^*−*/*−*^*/Apoer2*^+/+^ (N = 25,522 calls) and *Vldlr*^*−*/*−*^*/Apoer2*^*−*/*−*^ mice (N = 2,946 calls) compared to *Vldlr*^+/+^*/Apoer2*^+/+^ mice (Fig. 5B). There was a significant increase in the upward call type in the P7 *Vldlr*^*−*/*−*^*/Apoer2*^+/+^ group only, which parallels an increase in upward call type observed in *Dab1*^+/*lacZ*^ pups. No significant differences were found for the other call types. Thus, at P7 the *Vldlr*^*−*/*−*^*/Apoer2*^+/+^ pups had altered calling behavior reminiscent of that observed in *Dab1*^+/*lacZ*^ heterozygotes; and extremely restricted repertoires were observed in both *Vldlr*^*−*/*−*^*/Apoer2*^*−*/*−*^ and *Dab1*^*lacZ*/*lacZ*^ pups.

At P14, repertoire analysis revealed significant differences based on *Vldlr* genotype (Fig. 6; *Vldlr*^+/+^*/Apoer2*^+/+^, N = 1,021 calls, *Vldlr*^*−*/*−*^*/Apoer2*^+/+^, N = 3,360 calls, *Vldlr*^*−*/*−*^*/Apoer2*^*−*/*−*^, N = 1,554 calls). *Vldlr*^+/+^*/Apoer2*^+/+^ mice emitted the double call type significantly less often than *Vldlr*^*−*/*−*^*/Apoer2*^+/+^ and *Vldlr*^*−*/*−*^*/Apoer2*^*−*/*−*^ mice, and the short call type less than *Vldlr*^*−*/*−*^*/Apoer2*^*−*/*−*^ mice. Miscellaneous call types were significantly expanded in the *Vldlr*^*−*/*−*^*/Apoer2*^*−*/*−*^ compared to the *Vldlr*^*−*/*−*^*/Apoer2*^+/+^ pups. These findings reflect an extremely restricted repertoire of the *Vldlr*^*−*/*−*^*/Apoer2*^*−*/*−*^ at P14 as was shown in these animals at P7. This was also true of the single receptor mutants, albeit to a lesser degree. Thus, absence of *Vldlr*, and *Vldlr* with *Apoer2*, had a significant effect on calling repertoire at P7 and P14.

### Parallel effects of *Dab1* and *Vldlr* genotypes on repertoire correlation and syntax similarity

**Figure 7:**
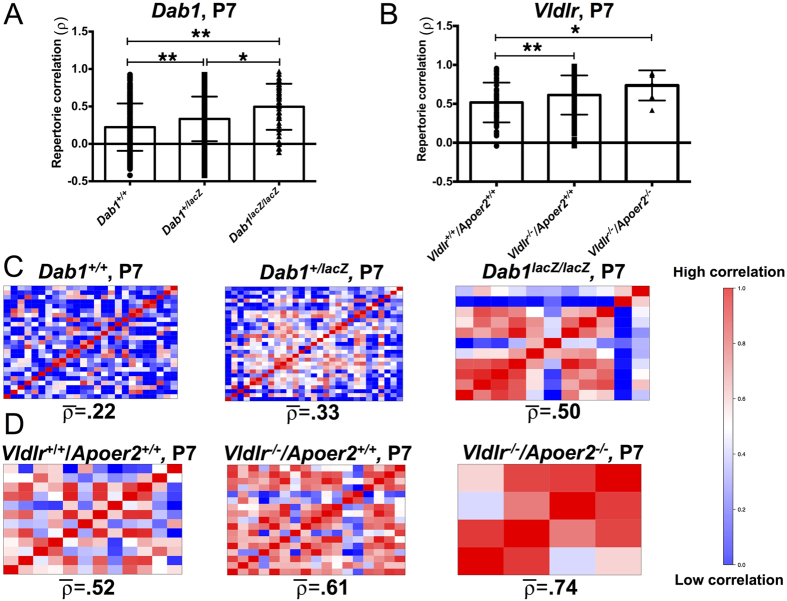
*Dab1* and *Vldlr*/*Apoer2* pups exhibit a gene-dose dependent increase in repertoire correlation at P7. (**A,B**) Repertoire correlation scores across all animals of each genotype. *Dab1*^+/*lacZ*^ pups have higher scores, reflecting a more restricted repertoire than *Dab1*^+/+^ pups (**p < *0.0001*). *Dab1*^*lacZ*/*lacZ*^ have higher scores than either *Dab1*^+/*lacZ*^ (*p = *0.0005*) or *Dab1*^+/+^ pups (**p < *0.0001*). *Vldlr*^*−*/*−*^*/Apoer2*^+/+^ have higher scores than *Vldlr*^+/+^*/Apoer2*^+/+^ (**p = *0.0131*), and *Vldlr*^*−*/*−*^*/Apoer2*^*−*/*−*^ pups exhibit a higher repertoire correlation than *Vldlr*^+/+^*/Apoer2*^+/+^ pups (*p = *0.038*). This pattern of increasing repertoire correlation in gene reduced or deficient pups is parallel across both lines (*Dab1, Vldlr/Apoer2*). (**C,D**) Repertoire correlation matrices for *Dab1* and *Vldlr/Apoer2* mice. Average repertoire correlation score is shown below each matrix (rho). Red indicates high correlation, and blue indicates low correlation on a scale of 0–1.

Repertoire correlation analysis of all pups was performed at P7 (Fig. 7A,B) to provide a measure of how similar individual repertories are within a given genotype. A high correlation between repertoires (positive correlation values, denoted by red) indicates convergence on similar call type usage, while a low correlation (negative correlation values, denoted in blue) are indicative of very different call usage between individuals (Fig. 7C,D). Overall, Dab1 mice exhibited a gene-dose dependent effect on repertoire correlation (one-way ANOVA, *p* < *0.001*). Pups with the *Dab1*^+/+^ genotype had the lowest repertoire correlation (average ρ = 0.22), followed by *Dab1*^+/*lacZ*^ (average ρ = 0.33, t-test *p* < *0.001*), then *Dab1*^*lacZ*/*lacZ*^ with the highest (average ρ = 0.50, t-test, *p* = *0.005*). The same was true for pups of the *Vldlr/Apoer2* genotype (one-way ANOVA, p = 0.015) with *Vldlr*^+/+^*/Apoer2*^+/+^ having lowest correlation scores (average ρ = 0.52), followed by the *Vldlr*^*−*/*−*^*/Apoer2*^+/+^ (average ρ = 0.61, t-test, *p* = *0.013*)); and then *Vldlr*^*−*/*−*^*/Apoer2*^*−*/*−*^ (average ρ = 0.74, *p* = *0.185*, not significant). Repertoire correlations of *Vldlr*^*−*/*−*^*/Apoer2*^*−*/*−*^ pups were significantly lower than those of *Vldlr*^+/+^*/Apoer2*^+/+^ pups (t-test *p* = *0.038*). These findings indicate an association between highly similar repertoires within the groups of low or no Reelin-signaling pathway components, i.e. *Dab1, Vldlr*, and *Apoer2*. Convergence on similar call types within a genotype would explain increasing repertoire correlation, and was most striking in the *Dab1*^*lacZ*/*lacZ*^ and *Vldlr*^*−*/*−*^*/Apoer2*^*−*/*−*^ pups.

**Figure 8:**
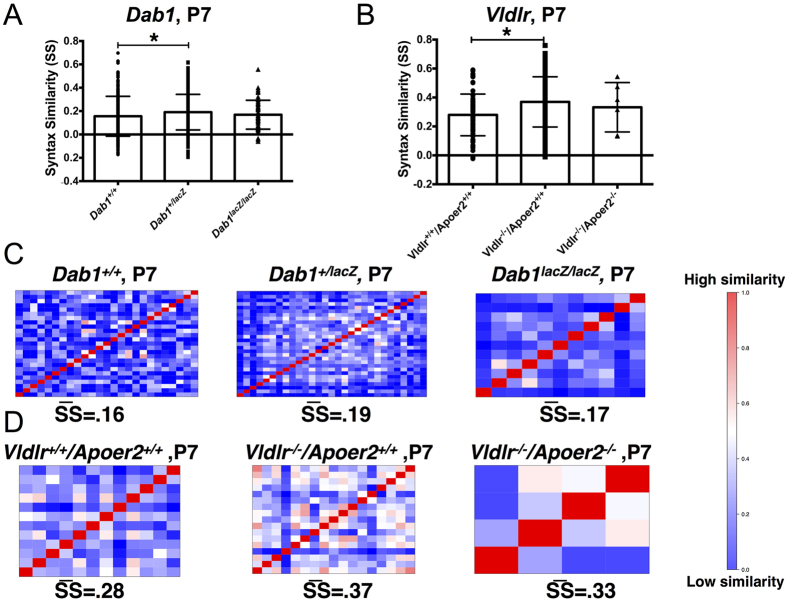
*Dab1*^+/*lacZ*^ and *Vldlr*^−/−^/*Apoer2*^+/+^ pups have high syntax similarity scores. (**A,B**) Syntax similarity scores across all animals of each genotype. *Dab1*^+/*lacZ*^ pups have higher scores than *Dab1*^+/+^ pups (*p = 0.005). There is no detectable difference between *Dab1*^*lacZ*/*lacZ*^ and *Dab1*^+/+^ pups. *Vldlr*^*−*/*−*^*/Apoer2*^+/+^ pups have higher scores than *Vldlr*^+/+^*/Apoer2*^+/+^ (**p = 0.0002). *Vldlr*^*−*/*−*^*/Apoer2*^*−*/*−*^ mice do not differ from *Vldlr*^+/+^*/Apoer2*^+/+^. (**C,D**) Syntax similarity matrices for *Dab1* and *Vldlr/Apoer2* mice. Average repertoire correlation score is shown below each matrix (SS). Red indicates high correlation, and blue indicates low correlation on a scale of 0–1.

The effect of genotype on call sequence, or syntax, was then examined using syntax similarity analysis of isolation calls for P7 pups (Fig. 8). This type of analysis shows how alike call transitions are between animals within a given group. High syntax similarity indicates similar types of transitions within a group. There was a significant effect of *Dab1* genotype on syntax similarity (Fig. 8A, one-way ANOVA, *p* = *0.014*). Syntax similarity was highest for the *Dab1*^+/*lacZ*^ *mice* (Syntax similarity average, SS = 0.19) compared to *Dab1*^*lacZ*/*lacZ*^ (SS = 0.17) and *Dab1*^+/+^ pups (SS = 0.16). Surprisingly, the *Dab1*^*lacZ*/*lacZ*^ pups had SS scores that were almost identical to that of *Dab1*^+/+^ pups. There was an effect of *Vldlr* genotype on SS as well (Fig. 8B, one-way ANOVA *p* = *0.002*). *Vldlr*^*−*/*−*^*/Apoer2*^+/+^ pups had higher similarity (SS = 0.37) than *Vldlr*^+/+^*/Apoer2*^+/+^ pups (SS = 0.28) and *Vldlr*^*−*/*−*^*/Apoer2*^*−*/*−*^ pups (SS = 0.33). In summary, parallel patterns of syntax similarity were observed across both the *Dab1* and *Vldlr/Apoer2* mouse lines.

## Discussion

Altered isolation vocalizations are a hallmark of ASD-like early phenotype in mice^33^. In order to determine if *Dab1* or *Vldlr* insufficiency impacts patterns of early social communication, we characterized the age related calling patterns in *Dab1* and *Vldlr/Apoer2* deficient mice, generating novel findings. Additionally, we queried whether or not the canonical Reelin-signaling pathway may be responsible for the changes in vocalization seen in *Reln*^+/−^ and *Reln*^*−*/*−*^ pups^57^,^62^. Despite extreme inter-individual variation in calling, we found that the *Dab1* genotype profoundly affected the calling rate and repertoire of P7 pups in a gene-dose dependent manner. The effect subsided at P14 in concert with the typical overall decrease in calling amount. Our findings reflect an ASD-like communicative pattern: reduced calling amount, reduced variety in syllable usage, and parallel changes seen in *Reln*^+/−^ and *Reln*^*−*/*−*^ pups.

We examined the effect of *Vldlr* deficiency on vocal phenotype, based on our previous findings which highlighted *Vldlr* as being vocally regulated in the basal ganglia of adult male zebra finches^64^. Changes in other genes that are critical for birdsong learning, including *Cntnp2* and *FoxP2*, have produced abnormal vocal communication patterns in neonatal mice^40^,^41^,^66^,^67^,^68^,^69^. These findings underscore shared mechanisms between vocal learning and non-learning species, and validate a cross-species approach. We found that *Vldlr*^*−*/*−*^ genotype alone did not affect calling rate in mice, but did significantly affect call repertoire at both time points. These changes were observed in both *Vldlr*^*−*/*−*^*/Apoer2*^+/+^ and *Vldlr*^*−*/*−*^*/Apoer2*^*−*/*−*^ groups indicating that loss of *Vldlr* is sufficient to produce these changes to the vocal repertoire. This limited syllable usage reflects a subtle ASD-like phenotype. The early vocal behavior of *Vldlr* insufficient pups had not been previously characterized.

Both *Dab1* and *Vldlr* gene dose affected the diversity of call repertoires, resulting in simpler call types (no frequency modulation, short duration) with fewer elaborate calls (jump containing, harmonic stacking, long duration). Parallels between the two mouse lines are further underscored by similarities in both the repertoire correlation and the syntax similarity measures. The more repetitive or stereotyped sequencing in both the *Dab1*^+/*lacZ*^ and *Vldlr*^*−*/*−*^*/Apoer2*^+/+^ lines may reflect a subtle vocal phenotype not uncovered by call count and repertoire analyses. The genetic changes in these lines are very different and thus convergence on a high degree of syntax similarity was not predicted. In auditory playback experiments, adult female mice prefer greater call complexity from both adult males and neonates^36^,^70^. It would therefore be advantageous for pups to emit more elaborate call types in order to be retrieved and thus survive. The restricted repertoire and convergence on simple syllable usage seen here in *Dab1* and *Vldlr* deficient pups would thus be maladaptive, as is the reduction in calling rate as dams prefer to retrieve pups that call more^35^.

Sex is another factor contributing to ASD etiology. Because ASD is more prevalent in males, we characterized early vocal phenotypes in each sex and compared them, expecting an exacerbated phenotype in males. To our surprise, when pooling across wild-type controls of both lines, there was no sex difference in calling rate or repertoire. Some minimal sex differences in repertoire were observed in *Dab1* and *Vldlr* deficient pups at P7, but none that suggested that one sex was more adversely affected by the gene loss of *Dab1* or *Vldlr* than the other. Thus, sex does not appear to interact with *Dab1* or *Vldlr/Apoer2* genotype to produce a more pronounced vocal phenotype. Prior studies provide conflicting reports regarding sex differences in the calling behavior of rodents with some indicating that male neonatal rats and mice call more, or that female mice do, or that there is no difference^71^. These disparate findings indicate that each species and strain should be individually tested rather than generalizing between studies regarding the influence of sex on vocal communication.

Loss of neonatal call type diversity is associated with reduced Reelin signaling as demonstrated here and in prior work. *Reln*^+/−^ *and Reln*^*−*/*−*^ pups on a similar background as used here, Romano and colleagues^62^ observed increased usage of two-syllable call type, and reduced numbers of short and flat call types with increasing *Reelin* insufficiency. In our study, we likewise observe an expansion of some call types and a reduction in others. While the exact call types differed, in both studies, increasingly restricted repertoires emerged in a gene-dose dependent manner. This similar gene-dose restriction across *Reelin, Dab1,* and *Vldlr/Apoer2* lines indicates a newly discovered function of the canonical Reelin-signaling pathway in shaping call-type usage.

ASD is a neurodevelopmental disorder in humans, and diagnosis is based, in part, on altered developmental trajectories and unusual social communication patterns^28^*. Reln*^+/−^ *and Reln*^*−*/*−*^ mouse pups exhibit differential vocal developmental trajectories; *Reln*^+/−^ pups have a delayed peak in calling and *Reln*^*−*/*−*^ pups lack a peak in calling^62^. We observed similarly altered trajectories for *Dab1*^+/*lacZ*^*, Dab1*^*lacZ*/*lacZ*^ *and Vldlr*^*−*/*−*^*Apoer2*^*−*/*−*^ mice. These findings also suggest that, like *Reln*^+/−^ mice^47^, *Dab1* insufficient mice may serve as a good ASD-risk mouse model. Future studies could determine whether or not these mice exhibit additional ASD-like behavioral features including repetitive behavior, decreased sociability, or behavioral inflexibility as adults. Once more is understood about the cellular phenotypes underlying Reelin signaling in the basal ganglia, targeted *Dab1* knock-out mice could be used to determine if a vocal phenotype is still present.

Building on previous work^62^, our findings identify a new role for the Reelin-signaling pathway in early vocal phenotypes in mice. It is noteworthy that any differences at all were observed in calling phenotype considering the high degree of inter-individual differences, particularly in call repertoire, that typify these vocalizations. Moreover, mouse pups congenitally engineered to lack a neocortex and hippocampus have indistinguishable calling patterns from wild-type pups^72^, emphasizing the significance of the deficits observed here. Since the lack of a cortex does not lead to abnormal calling, the deficits observed here may arise from alterations in subcortical structures. Notably, the basal ganglia has an established role in vocal learning^73^, cortico-striatal plasticity is altered in Reelin insufficient mice^50^, and abnormal basal ganglia connectivity and excitability are associated with ASD^55^,^56^. Together these observations provide a relevant yet understudied anatomical locus for future determination of the Reelin-associated neurodevelopmental mechanisms behind early vocal phenotypes.

## Methods

### Mouse breeding and care

Experiments were approved by UCLA Office of Animal Research Oversight. All animal use was in accordance with the UCLA Institutional Animal Care and Use Committee and complied with American Veterinary Association standards for working with laboratory animals. Mice were maintained on a 12 h light/dark cycle, with *ad libitum* food and water. *Dab1*^*lacZ*^ mice were a gift from Dr. Brian Howell (Upstate Medical University, SUNY). The mice have a truncation of Dab1 at residue 22 and expression of a fusion of the *lacZ* reporter rendering the protein unable to initiate downstream signaling via phosphorylation at critical residues^66^. These mice were generated as previously described^74^ by breeding *Dab1 cKIneo* mice with Meox-Cre germline deleter mice (B6.129S4-*Meox2*^*tm1*(*cre*)*SOR*^/J, Jackson Laboratories, Bar Harbor, ME, USA) and bred into B6;129Sv. The expression of beta-galactosidase is in line with established Dab1 expression in cerebral cortex, cerebellum, and hippocampus^74^,^75^. *Vldlr*^*tm1Her*^ mice have a targeted complete deletion of the *Vldlr* gene and were generated in B6;129S7 mice^76^. Double receptor mutants do not have Apoer2 or Vldlr and resemble *Reln*^*−*/*−*^ mice^13^. *Dab1, Vldlr,* and *Apoer2* mice were genotyped using PCR as previously described^74^,^76^,^77^.

### Vocal Recording

To test isolation calls, mouse pups were removed from the nest, four at a time, and individually placed into sound attenuation chambers for recording. These chambers were constructed from small coolers (Coleman) that were coated inside with soundproof foam (Soundcoat). An ultrasonic microphone (UltraSoundGate, Avisoft Bioacoustics) was suspended above the pup. Recordings were conducted at P7 and P14 for a total of 15 minutes at each time point. In order to not affect calling patterns of any remaining pups in a given litter, only four (the total number of recording chambers) from each litter were recorded. After recording at P7, pups were tailed for genotyping, and tattooed to enable identification for re-recording at P14. The initial distance between the microphones and the pups was equivalent across chambers at P7 when pups are fairly immobile. At P14, pups are ambulatory so their distance from the microphone varied. Because of this, amplitude measurements were not included in the acoustic analysis. Pups were recorded within the same 2-hour time window each day (light: 14:00–16:00 hr) to avoid circadian effects. Temperature was maintained at 21–22 °C.

### Acoustic analysis, quantification and classification

Ultrasonic (20–125 kHz) vocalizations were acquired using a sampling rate of 250 kHz. In order to reduce background noise and focus on ultrasound, sounds with a frequency <40 kHz were high-pass filtered and removed from analysis. Recorded vocalizations were segmented based on amplitude threshold to allow for recording of bouts (Avisoft-SASLab Pro Recorder). Bouts are a series of USVs that occur in rapid succession (<40 ms between calls) and are surrounded by >1 second of silence. Recordings were transduced from amplitude traces into spectrograms using Fast Fourier Transform with a transform of 256 points and a time window overlap of 75% (Avisoft Bioacoustics; SASLab Pro). Bouts were then segmented into individual syllables and then processed using VoICE, a semi-automated unbiased clustering mechanism, to classify these calls into categories^66^.

Call type categories from the work of Scattoni and colleagues^45^ were used and include 9 basic types: ‘short’ (duration <10 ms), ‘downward’ (frequency sweeps downward of >10 kHz and >10 ms), ‘upward’ (frequency sweeps upward of >10 kHz, >10 ms), ‘flat’ (<10 kHz of modulation, >10 ms), ‘complex’ (wave shaped frequency sweep, >10 ms) ‘frequency step’ (multiple jump containing calls), ‘chevron’ (inverted U shape frequency with >10 kH of modulation), ‘harmonic’ (multiple jump containing with harmonic stacking), and ‘two-syllable’ (one-jump containing). Composite call types (those containing no jumps but with harmonic stacking) were collapsed into the harmonic call category; unstructured call types (broadband of >40 kHz with no clear single frequency) comprised <1% of the recordings and were not analyzed. Additional call categories of ‘doubles’, ‘triples’, and ‘miscellaneous’ were observed and included. Double and triple calls are comprised of various frequency sweeps that occur in rapid succession, being separated by <10 ms. These were rare and considered together as a single call type. Miscellaneous call types did not fit into any of the groups described previously, and may represent emerging novel types. The sequence of the calls, referred to here as ‘syntax’, was also assessed. Syntax similarity, syntax entropy and repertoire correlation analyses were performed as described previously^66^.

### Statistical methods

Where possible, resampling statistical tests were used because this methodology makes no assumptions about the data distribution. Call counts were quantified and analyzed using two-way ANOVA followed by individual 2-tailed t-tests with as follows: Once call classifications were determined, we noticed a high degree of variability between individual pups of the same genotype (Supplemental Fig. 1). To overcome this variability, we normalized each raw call count in each category to the total number of call counts per animal. These normalized values were used to create pie charts. In order to assess statistical differences in repertoire between groups, call count categories of each animal were rank transformed and then resampled 10,000 times to determine the median rank and 95% confidence interval (CI) for each genotype. Only measures with non-over lapping CIs were considered to differ. Syntax similarity scores, syntax entropy scores, and repertoire correlation were also subjected to one-way ANOVA followed by *post hoc* 2-tailed t-tests with Welch’s correction.

## Additional Information

**How to cite this article**: Fraley, E. R. *et al*. Mice with *Dab1* or *Vldlr* insufficiency exhibit abnormal neonatal vocalization patterns. *Sci. Rep.* **6**, 25807; doi: 10.1038/srep25807 (2016).

## Supplementary Material

Supplementary Information
