# Supplementary material for: Mice with Dab1 or Vldlr insufficiency exhibit abnormal neonatal vocalization patterns
Source: Sci Rep. 2016 May 17;6:25807. doi: 10.1038/srep25807 (PMC4868998; doi:10.1038/srep25807)
Supplement: Supplementary Information [file srep25807-s1.pdf]

Mice with *Dab1* or *Vldlr* insufficiency exhibit abnormal neonatal vocalization patterns

Fraley ER, Burkett ZB, Day NF, Schwartz BA, Phelps PE, White SA

Supplemental Figure 1. *Lack of substantial sex differences in calling behavior of Dab1 insufficient pups.* (A) Calling amount is not influenced by sex at P7 (left) or P14 (right) in *Dab1* pups. (B) Calling repertoire is not affected by sex in *Dab1*$^{+/lacz}$ pups at P7. Call type is plotted on the x axis, and the rank sum score median with 95% confidence intervals is plotted on the y axis. Circles represent males and squares represent females. All confidence intervals overlap indicating no differences in repertoire. (C) Calling repertoire is slightly impacted by sex in *Dab1*$^{lacZ/lacZ}$ pups at P7. Males emit fewer downward and two-syllable call types than do females.

Supplemental Figure 2. *Calling repertoires of all mice without normalization.* (A) Repertoire distribution across *Dab1* mice at P7. Stacked bar plots show all data from all pups recorded. The y axis represents raw call count, and the x axis represents call type. Each color represents an individual mouse. The far left shows data from the *Dab1*$^{+/+}$genotype, the middle, *Dab1*$^{+/lacZ}$, and the far right, *Dab1*$^{lacZ/lacZ}$. (B) Repertoire distribution across Dab1 mice at P14, organized by genotype as in (A). (C) Repertoire distribution across all Vldlr/Apoer2 mice at P7. The far left plot is for *Vldlr*$^{+/+}$*/Apoer2*$^{+/+}$, the middle is, *Vldlr*$^{-/-}$*/Apoer2*$^{+/+}$*,* and the far right is *Vldlr*$^{-/-}$*/Apoer2*$^{-/-}$ pups. (D) Repertoire distribution across all *Vldlr/Apoer2* mice at P14*,* as organized by genotype in (C).

Supplemental Figure 3. *No sex differences in calling behavior of wild-type mice at P7.* (A) Data from all wild-type mice (i.e. *Dab1*$^{+/+}$, *Vldlr*$^{+/+}$*/Apoer2*$^{+/+}$*)* in this study were pooled and analyzed. Call counts and calling repertoire (B) show no sex specific differences at P7.

Supplemental Figure 4. *Lack of substantial sex differences in calling behavior of Vldlr and or Apoer2 insufficient pups.* (A) *Calling rates by sex of Vldlr*$^{-/-}$*/Apoer2*$^{+/+}$ *and Vldlr*$^{-/-}$*/Apoer2*$^{-/-}$ *pups.* Plots display the amount of calling by genotype, separated by sex. No differences are apparent at P7, but at P14, *Vldlr*$^{-/-}$*/Apoer2*$^{+/+}$ males call less than females. (B) Differential calling repertoire of *Vldlr*$^{-/-}$*/Apoer2*$^{+/+}$ mice based on sex. Males of this genotype emit more short calls than do females. (D) Differential calling repertoire of *Vldlr*$^{-/-}$*/Apoer2*$^{-/-}$ mice based on sex. Males of this genotype emit more flat and double type calls than do females. Due to the low number of surviving animals in this group, resampling statistics do not provide large confidence intervals, leading to single points.

A

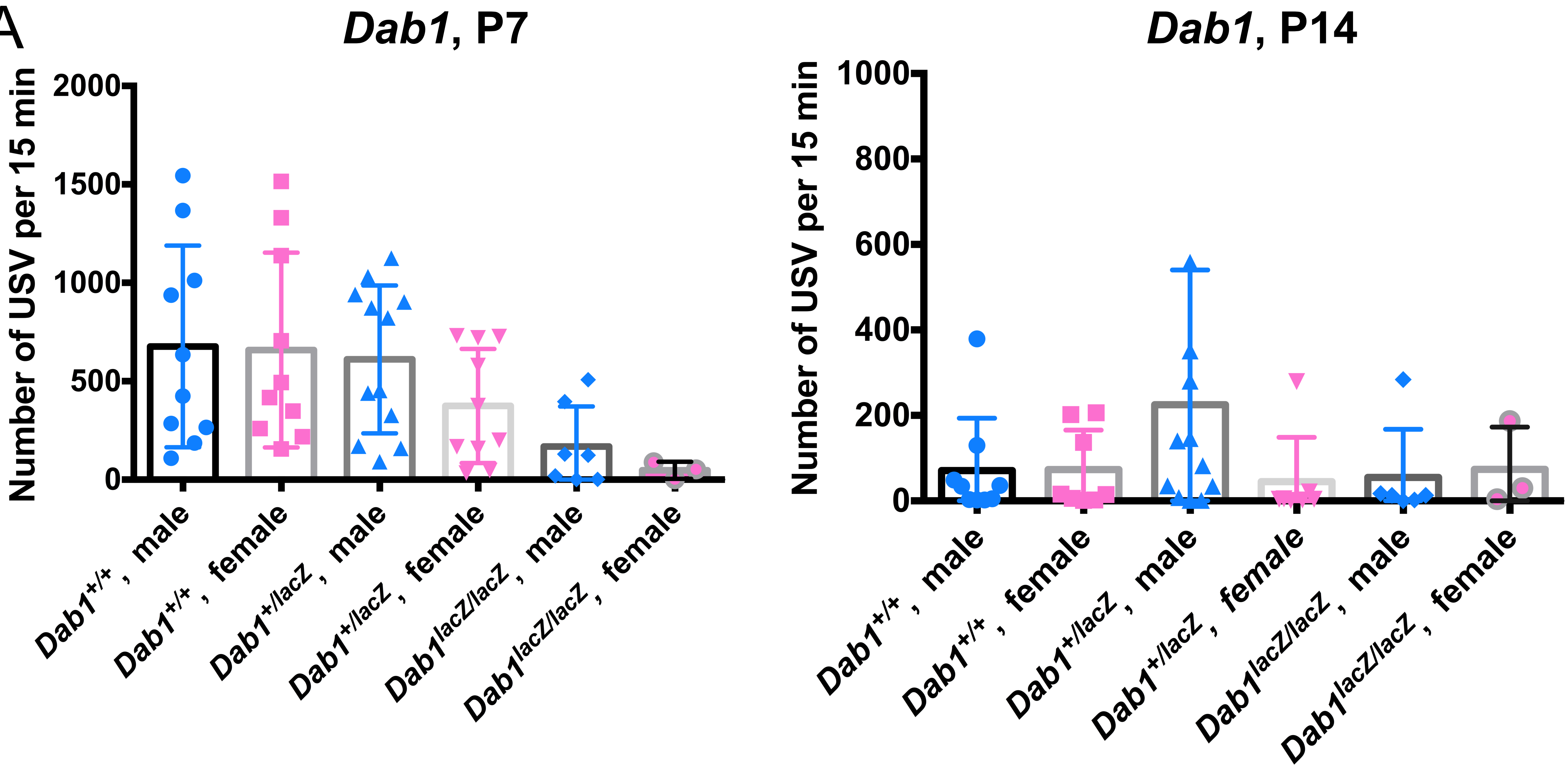

Dab1, P7
Number of USV per 15 min
2000
1500
1000
500
0
Dab1+/+, male
Dab1+/+, female
Dab1+/lacZ, male
Dab1+/lacZ, female
Dab1lacZ/lacZ, male
Dab1lacZ/lacZ, female
Dab1, P14
Number of USV per 15 min
1000
800
600
400
200
0
Dab1+/+, male
Dab1+/+, female
Dab1+/lacZ, male
Dab1+/lacZ, female
Dab1lacZ/lacZ, male
Dab1lacZ/lacZ, female

B

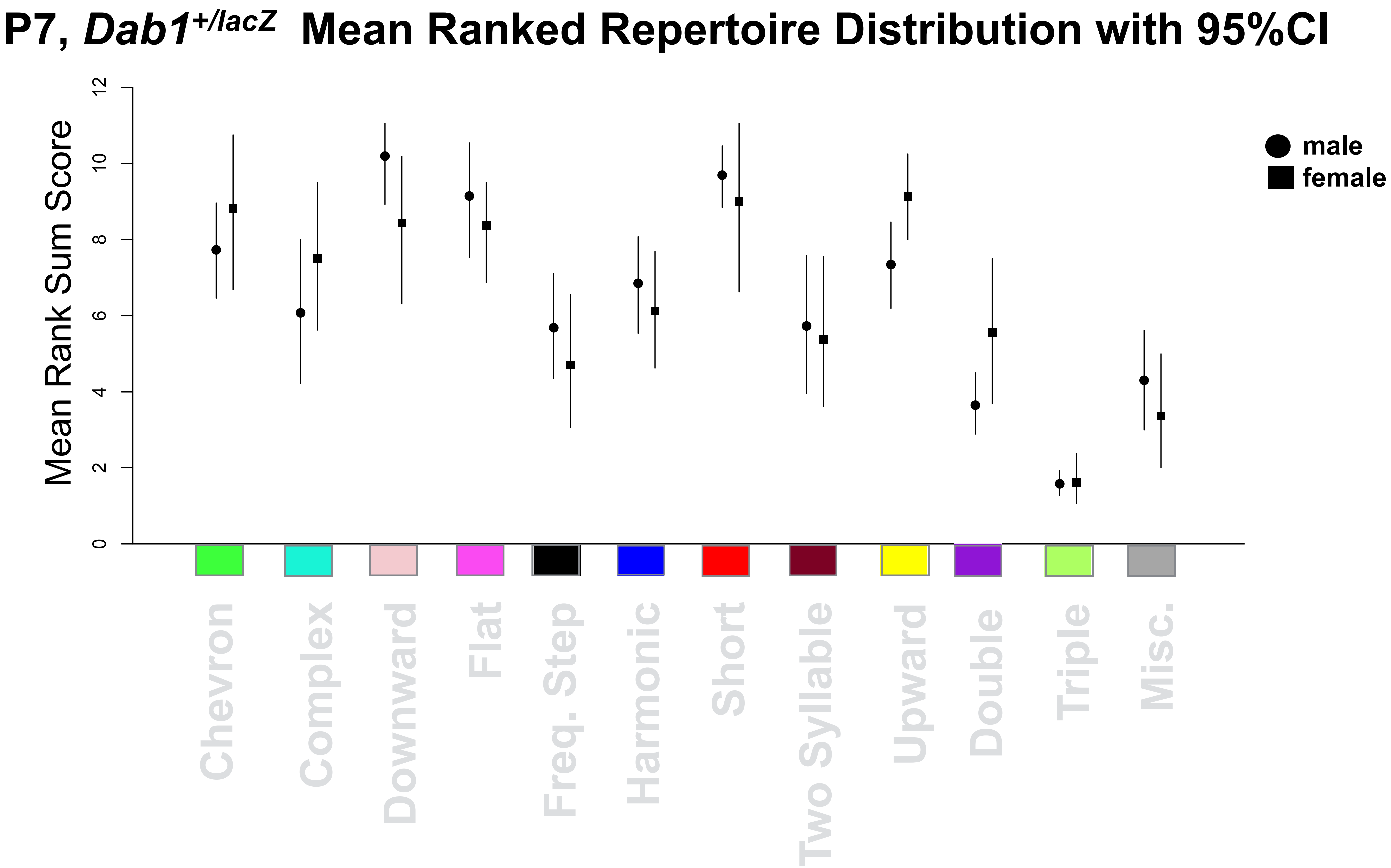
P7, *Dab1*$^{+/lacZ}$ Mean Ranked Repertoire Distribution with 95%CI
Mean Rank Sum Score
0
2
4
6
8
10
12
male
female
Chevron
Complex
Downward
Flat
Freq. Step
Harmonic
Short
Two Syllable
Upward
Double
Triple
Misc.

C

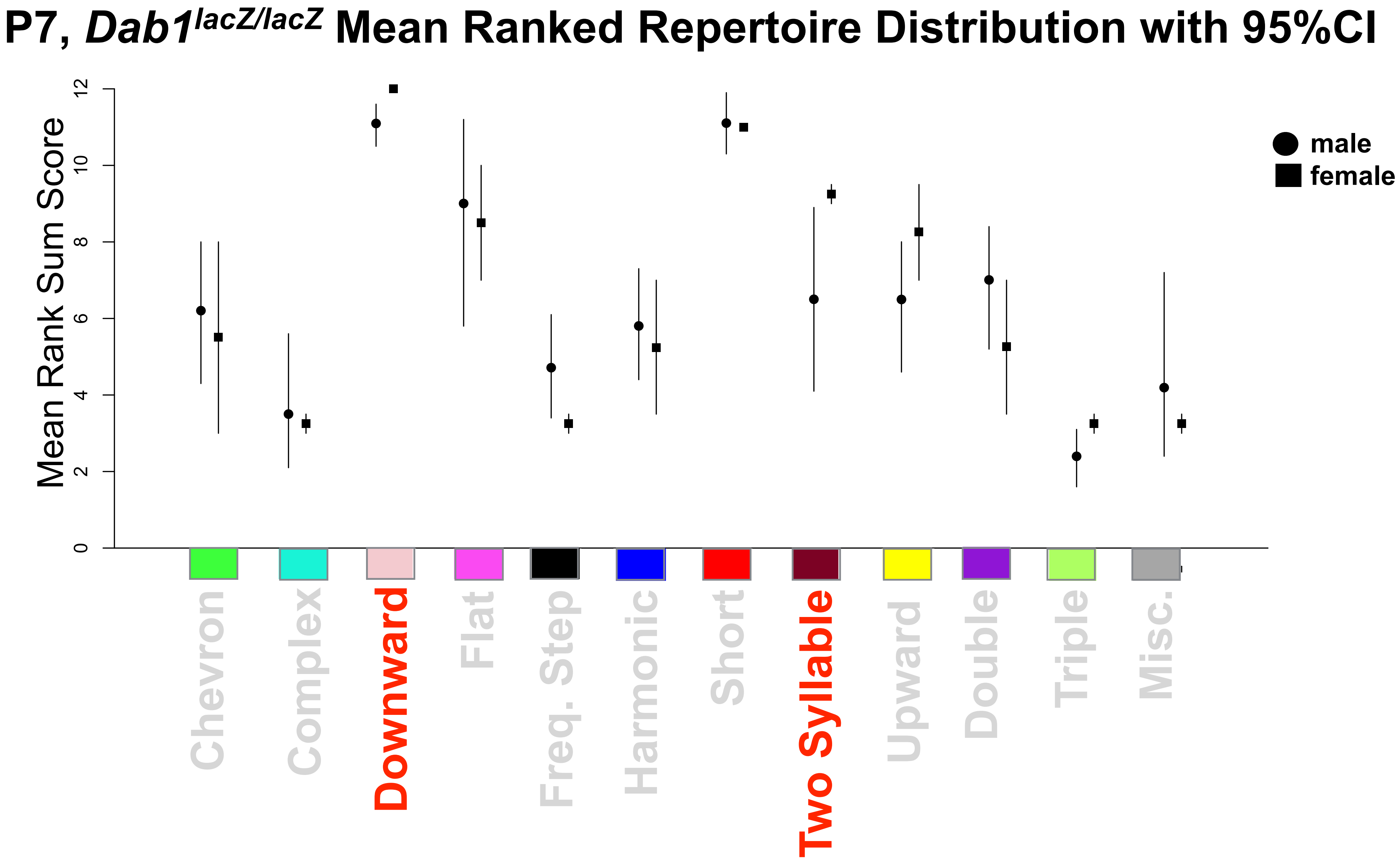

P7, Dab1lacZ/lacZ Mean Ranked Repertoire Distribution with 95%CI
Mean Rank Sum Score
0
2
4
6
8
10
12
male
female
Chevron
Complex
Downward
Flat
Freq. Step
Harmonic
Short
Two Syllable
Upward
Double
Triple
Misc.

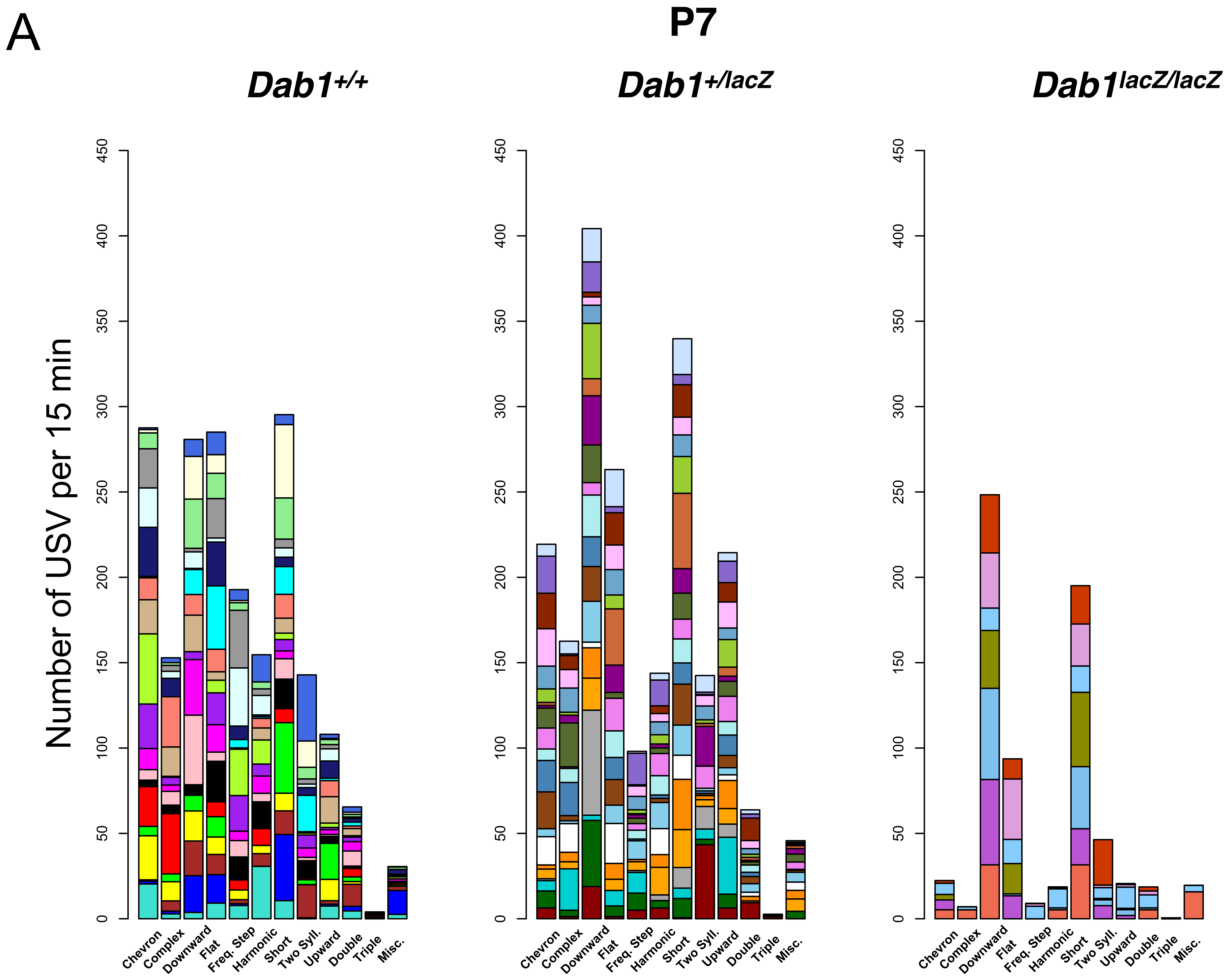
A
P7
Dab1+/+
Dab1+/lacZ
Dab1lacZ/lacZ
Number of USV per 15 min
0
50
100
150
200
250
300
350
400
450
Chevron
Complex
Downward
Flat
Freq. Step
Harmonic
Short
Two Syll.
Upward
Double
Triple
Misc.

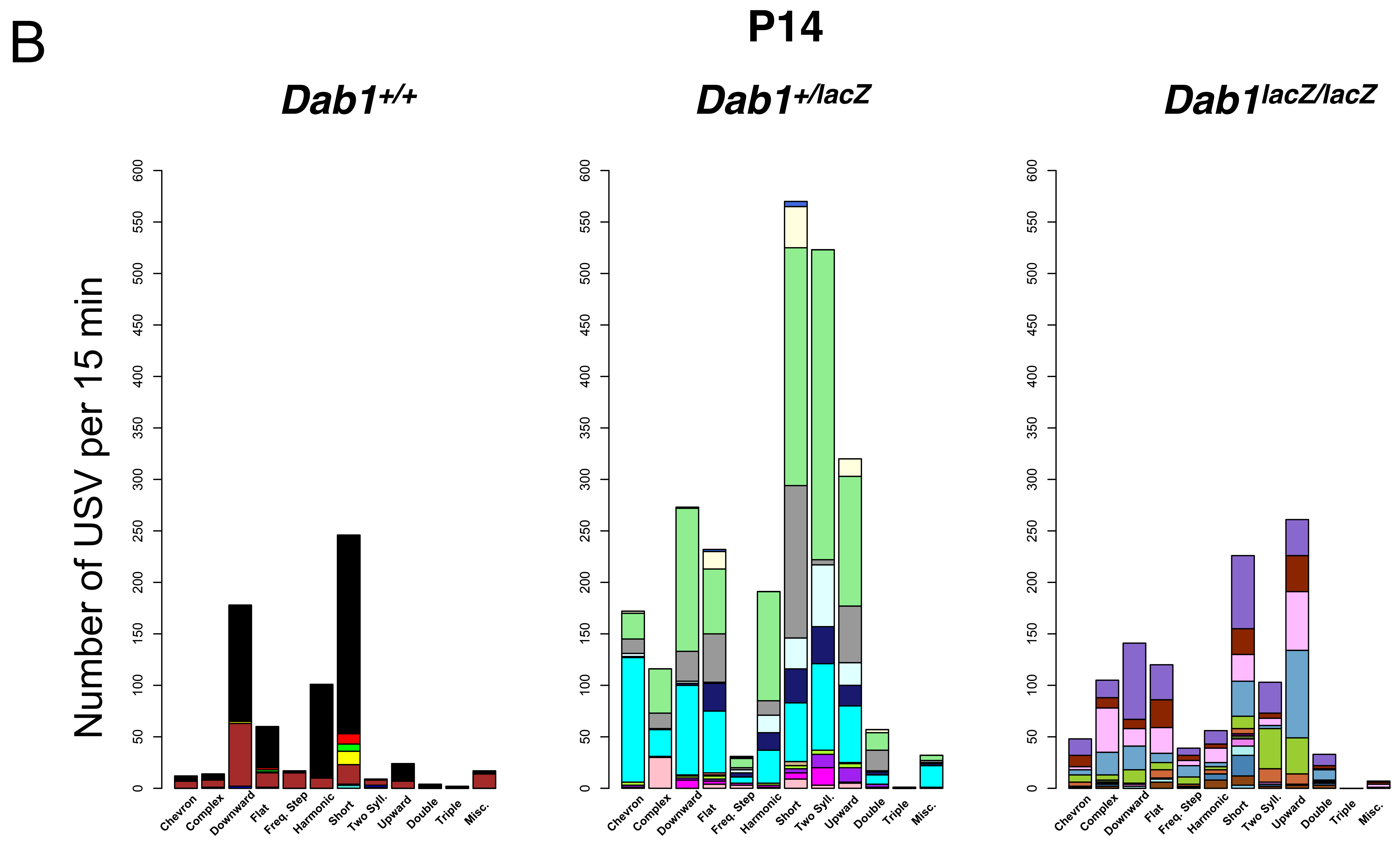

B
P14
Dab1+/+
Dab1+/lacZ
Dab1lacZ/lacZ
Number of USV per 15 min
0
50
100
150
200
250
300
350
400
450
500
550
600
Chevron
Complex
Downward
Flat
Freq. Step
Harmonic
Short
Two Syll.
Upward
Double
Triple
Misc.

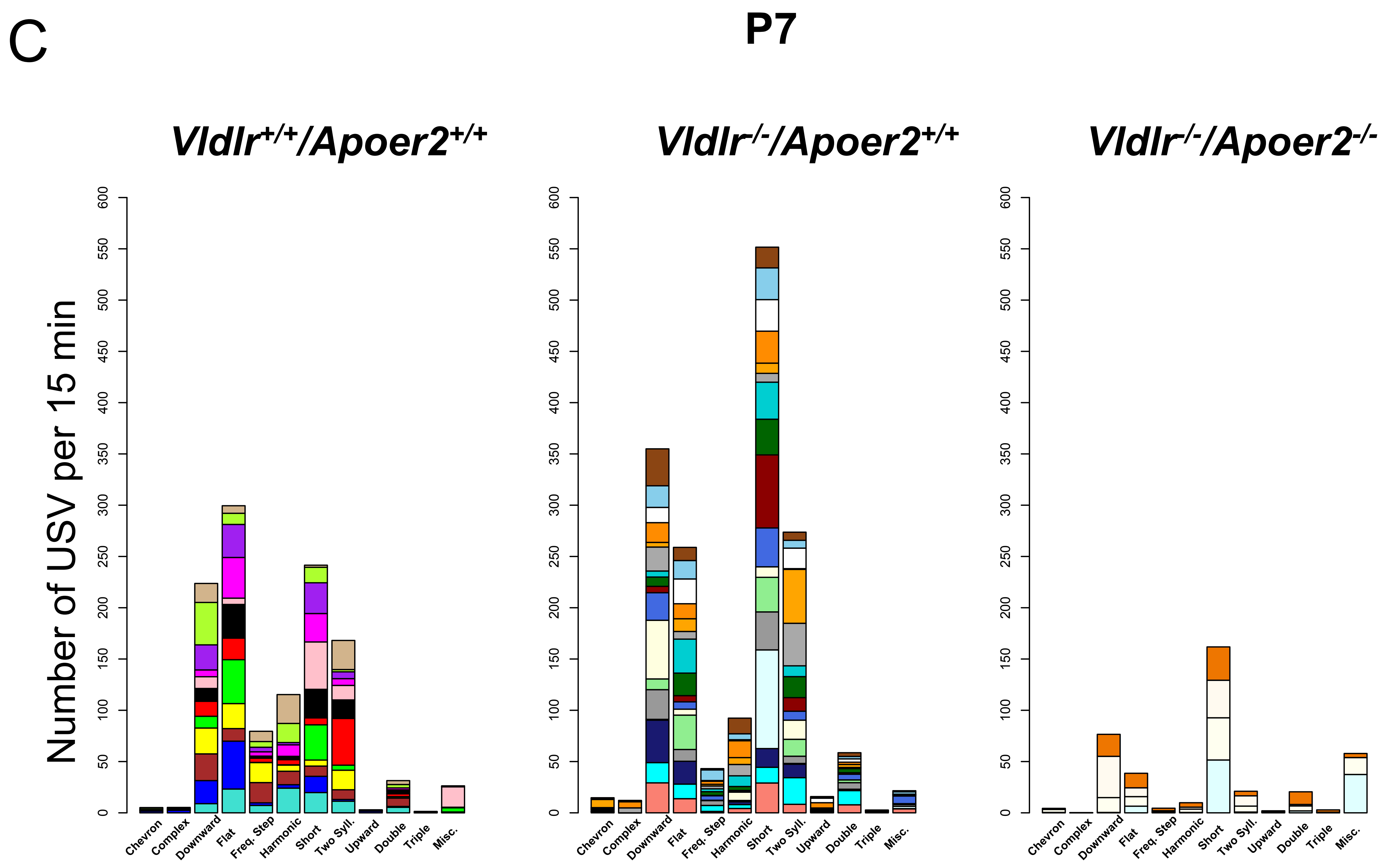
C
P7
Vldlr+/+/Apoer2+/+
Vldlr-/-/Apoer2+/+
Vldlr-/-/Apoer2-/-
Number of USV per 15 min
0
50
100
150
200
250
300
350
400
450
500
550
600
Chevron
Complex
Downward
Flat
Freq. Step
Harmonic
Short
Two Syll.
Upward
Double
Triple
Misc.

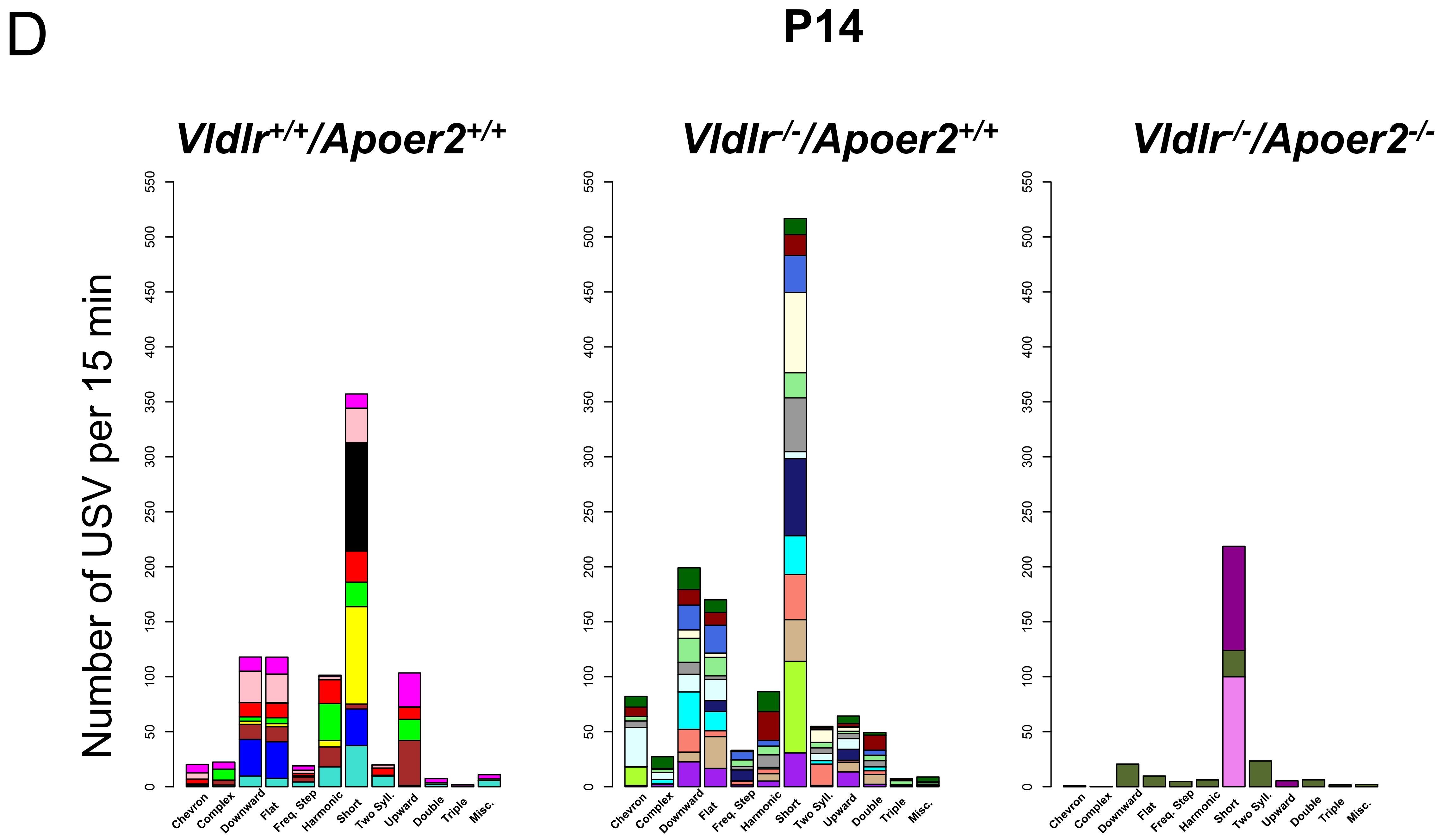

D
P14
Vldlr+/+/Apoer2+/+
Vldlr-/-/Apoer2+/+
Vldlr-/-/Apoer2-/-
Number of USV per 15 min
0
50
100
150
200
250
300
350
400
450
500
550
Chevron
Complex
Downward
Flat
Freq. Step
Harmonic
Short
Two Syll.
Upward
Double
Triple
Misc.

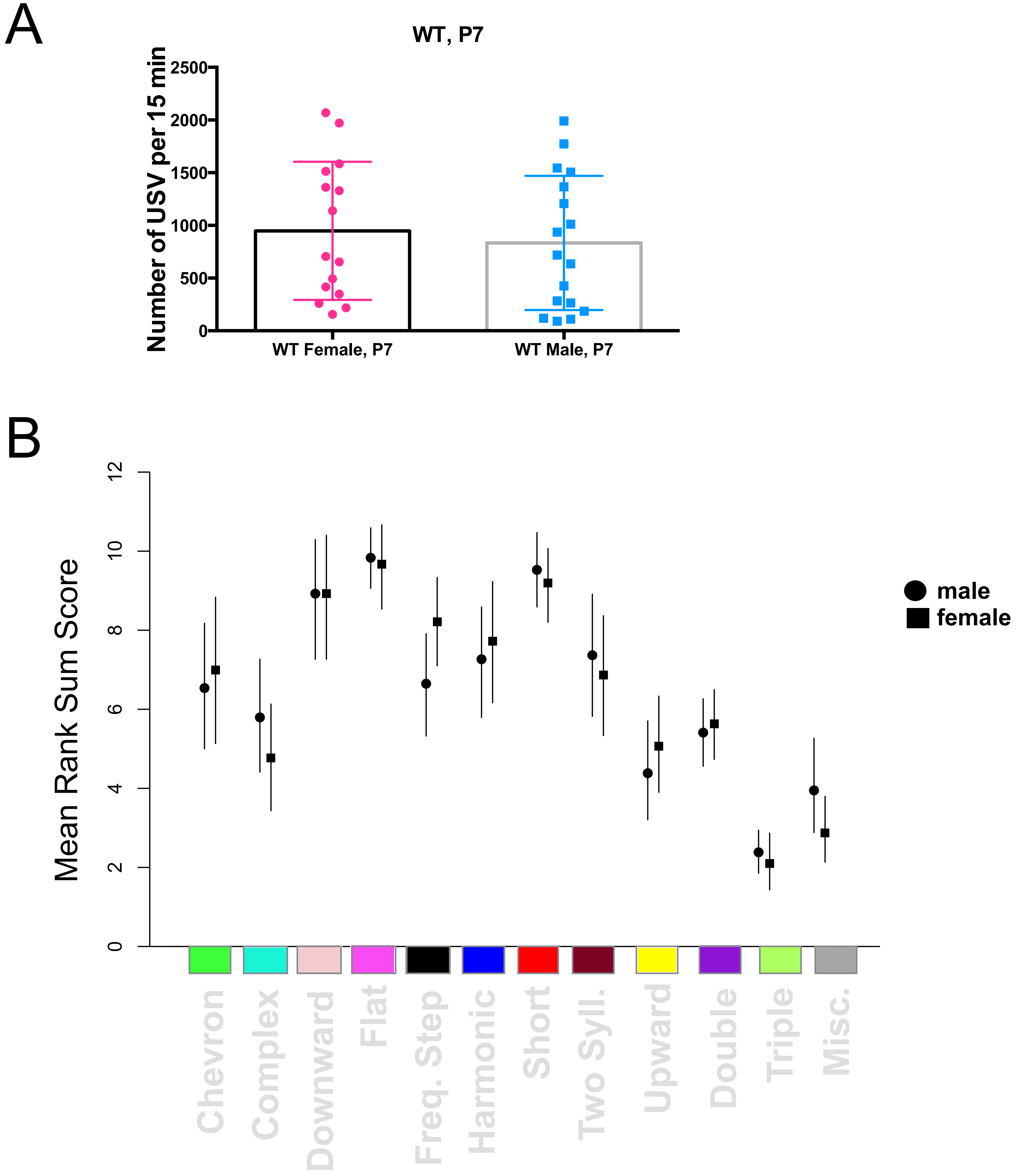
A
WT, P7
Number of USV per 15 min
2500
2000
1500
1000
500
0
WT Female, P7
WT Male, P7
B
Mean Rank Sum Score
12
10
8
6
4
2
0
male
female
Chevron
Complex
Downward
Flat
Freq. Step
Harmonic
Short
Two Syll.
Upward
Double
Triple
Misc.

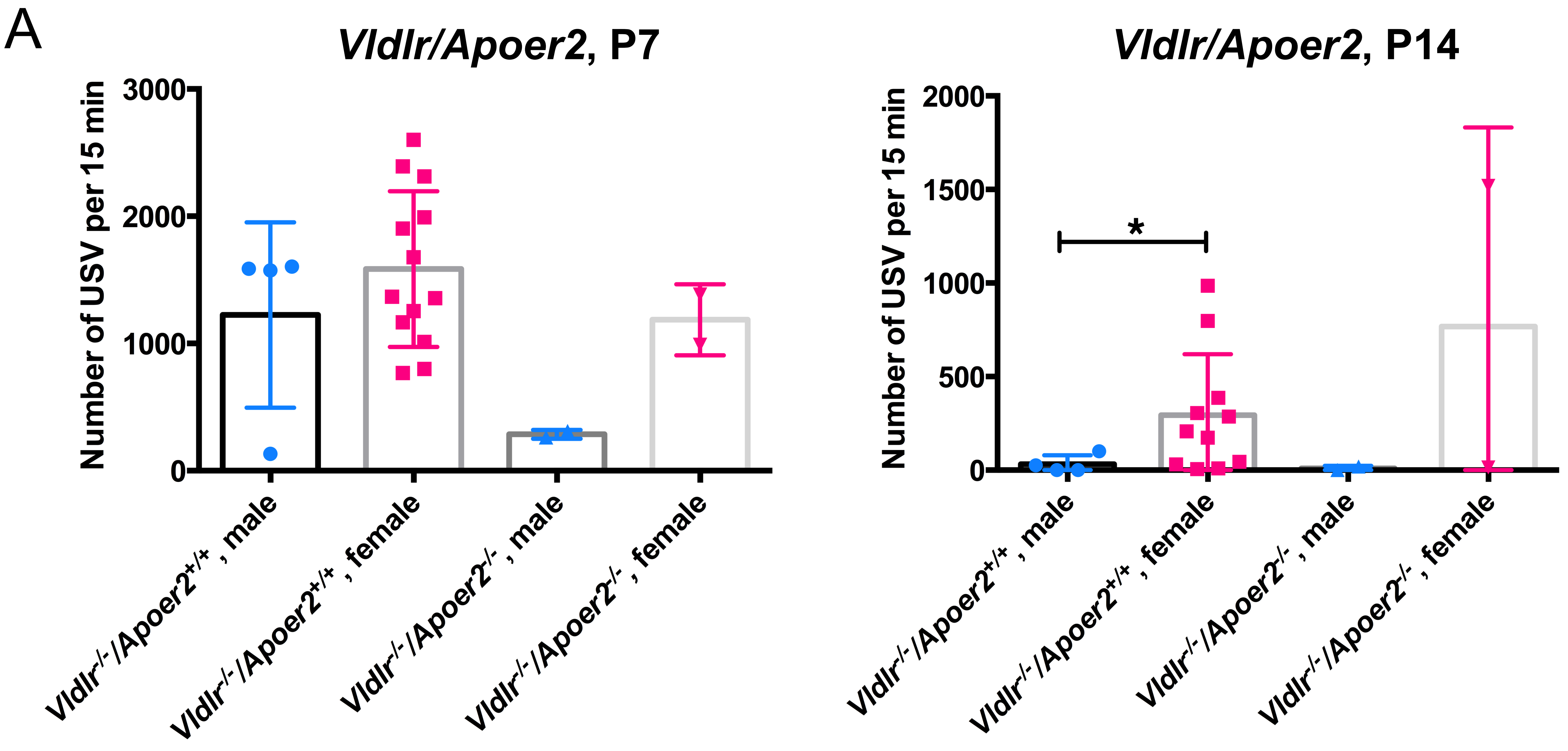

A
Vldlr/Apoer2, P7
Number of USV per 15 min
3000
2000
1000
0
Vldlr-/-/Apoer2+/+, male
Vldlr-/-/Apoer2+/+, female
Vldlr-/-/Apoer2-/-, male
Vldlr-/-/Apoer2-/-, female
Vldlr/Apoer2, P14
Number of USV per 15 min
2000
1500
1000
500
0
*
Vldlr-/-/Apoer2+/+, male
Vldlr-/-/Apoer2+/+, female
Vldlr-/-/Apoer2-/-, male
Vldlr-/-/Apoer2-/-, female

B

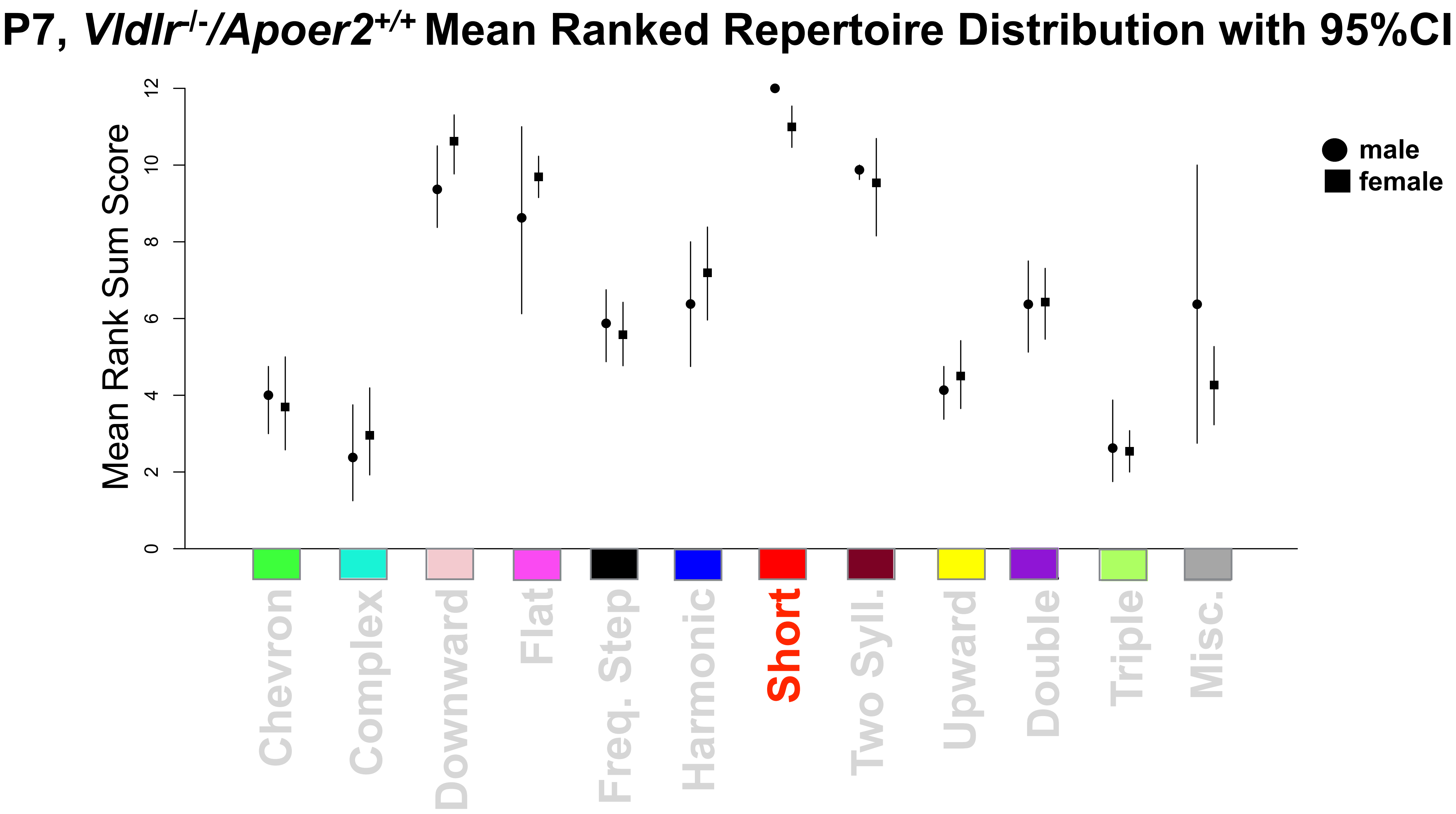

P7, Vldlr-/-/Apoer2+/+ Mean Ranked Repertoire Distribution with 95%CI
Mean Rank Sum Score
0
2
4
6
8
10
12
male
female
Chevron
Complex
Downward
Flat
Freq. Step
Harmonic
Short
Two Syll.
Upward
Double
Triple
Misc.

C

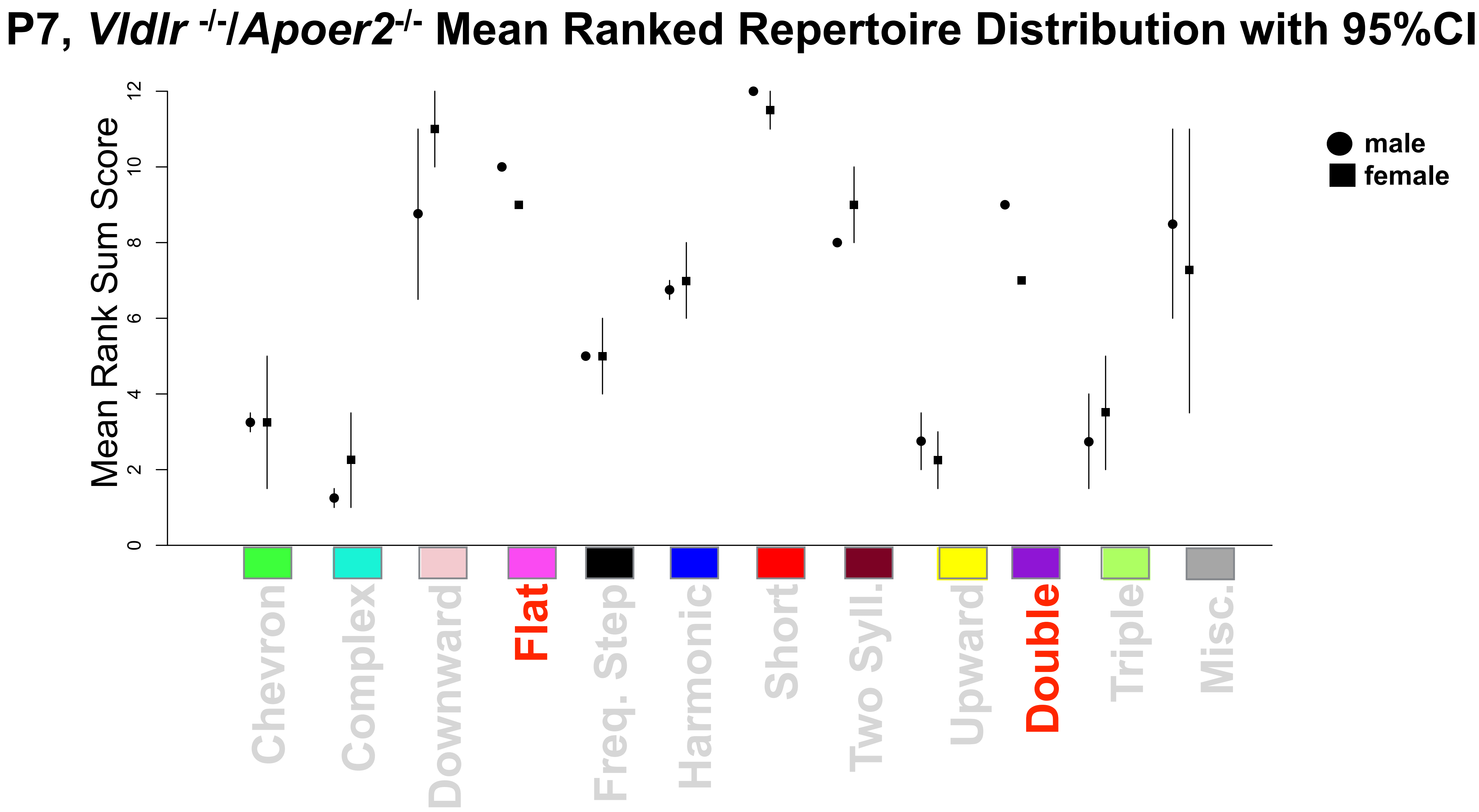
P7, Vldlr -/-/Apoer2-/- Mean Ranked Repertoire Distribution with 95%CI
Mean Rank Sum Score
0
2
4
6
8
10
12
male
female
Chevron
Complex
Downward
Flat
Freq. Step
Harmonic
Short
Two Syll.
Upward
Double
Triple
Misc.
